# Validation of the Benefits of Being a Caregiver Scale (BBCS) – further development of an independent characteristic of informal caregiving

**DOI:** 10.1186/s12877-022-03650-y

**Published:** 2023-01-14

**Authors:** Anna Pendergrass, Saskia Weiß, Nicolas Rohleder, Elmar Graessel

**Affiliations:** 1 Department of Psychiatry and Psychotherapy, Centre of Health Services Research in Medicine, Universitätsklinikum Erlangen, Friedrich-Alexander-Universität Erlangen-Nürnberg (FAU), Schwabachanlage 6, 91054 Erlangen, Germany; 2 German Alzheimer Society, Berlin, Germany; 3 Department of Psychology, Chair of Health Psychology, Friedrich-Alexander-Universität Erlangen-Nürnberg, Erlangen, Germany

**Keywords:** Positive aspects of caregiving, Benefits of Being a Caregiver Scale, Factor analysis, Reliability, Validity, Item difficulty, Discriminatory power

## Abstract

**Background:**

Although larger amounts of scientific attention have been directed toward the concept of positive aspects of caregiving (PAC) in recent years, a globally uniform definition and a suitable, scientifically valid questionnaire for all informal caregivers have yet to be developed. On the basis of the questionnaires that already exist for measuring PAC, the authors aimed to (a) concretize the concept and (b) develop a new scale by focusing only on items that show that family caregivers experience a benefit for themselves and that the benefit they experience is the result of their caregiving activities.

**Methods:**

The Benefits of Being a Caregiver Scale (BBCS) was validated on data from 961 informal caregivers. Cronbach's alpha was calculated to assess the internal consistency of the items, and a factor analysis was conducted to determine the structure of the BBCS. The discriminatory power and item difficulties were examined. Construct validity was established by testing four hypotheses.

**Results:**

The factor analysis confirmed the single-factor structure of the BBCS. Cronbach's alpha for the total scale was 0.922. One of the 15 items did not show good to very good discriminatory power and was excluded from the final version of the scale. A higher BBCS score was observed if the caregiver experienced more positive aspects of caregiving and tended to have better general coping skills and a positive relationship with the care-receiver. The BBCS score was not associated with the subjective burden of the caregiver. Results confirmed the validity of the BBCS.

**Conclusion:**

The BBCS is a valid assessment instrument for measuring the benefits that caregivers experience from their caregiving work and can easily be used in research and practice. The BBCS is available free of charge in English and German (http://www.caregiver-benefits.de).

## Introduction

Scientific research has been able to show that informal caregivers (CGs) experience not only negative but also positive effects from their caregiving work [1, 2]. The so-called positive aspects of caregiving (PAC) include, for example, the feeling of being needed [3], the appreciation experienced by the care-receiver [4], personal growth and maturity [5], learning new skills [6], and becoming a stronger and more resilient person in general [7]. Due to the high prevalence and the existence of PAC [8], the PAC construct has received a great deal of scientific attention in recent years. In Germany, for example, 87% of caregiving relatives stated that they have experienced PAC in at least one of the five areas surveyed by the subscale of the Berlin Inventory of Caregivers' Burden with Dementia Patients (BIZA) [6, 9].

Semiatin and O’Connor [10] provided important indications of the advantageous effects that PAC has on CGs, care-receivers (CRs), and the overall situation. They found evidence of a buffer effect of PAC. That is, negative psychological (e.g., depression) and physiological (e.g., physical discomfort) effects of caregiving could be mitigated by experiencing PAC. In addition, Schulz et al. [11] showed that people reporting more PAC tended to care for their relatives for longer, which meant that moving into a nursing home could be delayed.

The idea that PAC represents a much more complex concept than originally assumed was pointed out in studies by Lloyd et al. [12] and Sanders [13]. They showed that the original assumption that CG benefits and CG burden represent two extremes of the same continuum could be refuted. They demonstrated that co-experiencing negative and positive aspects of caregiving is possible and that different factors influence the experience of these two independent constructs. Therefore, future CG interventions should focus not only on reducing the burden on CGs but also on increasing CGs’ PAC to improve the entire care situation.

Due to varying conceptualizations, there are different ways to operationalize PAC (e.g. [2–7, 14–22]). This variability in operationalizations and the unclear underlying definitions may also account for the inconsistent results related to this construct and promotes theoretical confusion [23]. Furthermore, some items from the existing questionnaires are not specifically related to caregiving, cannot be influenced by an intervention, or are not formulated clearly (e.g. two different aspects are evaluated with a single item or the item can be interpreted as something positive or negative). Additionally, not all the questionnaires were developed with the participation of CGs, and most of the validation studies focused on one specific subpopulation (mainly CGs of people with dementia).

For example, the often-used 9-item Positive Aspects of Caregiving Scale (PACS) [3] from the United States was developed and validated in a sample of CGs of people with dementia and focuses on two different components of PAC: self-affirmation and outlook on life. Despite the scale’s two-factor structure, the items from the two factors are summed to generate a single outcome score. All items are related to caregiving, but the wording of some of the items is vague. For example, the item “Providing help/care to…has made me feel strong and confident” includes the facet of feeling strong and the facet of feeling confident. Thus, the item addresses two different facets that do not necessarily occur together.

The newer Spanish Gains Associated with Caregiving (GAC) Scale [5] also focuses on CGs of people with dementia by evaluating the gains that CGs experience from their role across five different domains: industry, identity, intimacy, generativity, and ego integrity. Although the items cover very important aspects of caregiving, some of the items are imprecise and can be interpreted in different ways, for example, “Being a caregiver has helped me to express more freely what I think or feel without offending other people.”

A subscale of the German BIZA scale [6] focuses on five different areas of positive aspects of caregiving (reconsideration, priorities, strength, learning experience, maturity). It is noticeable that the operationalization of some items do not clearly refer to a benefit, for example “I feel that because of my caregiving activities, I see many things differently than I used to” (from the reconsideration domain).

Although scientific attention directed toward the concept of PAC has been increasing in recent years, there is neither a globally uniform definition worldwide [12] nor a suitable, scientifically valid questionnaire for capturing the positive aspects of caregiving experienced by caregivers. With this article, we aim to concretize and "sharpen" the concept of "positive aspects" and to create a new scale that further develops the items from existing PAC questionnaires. To address the criticism of the lack of underlying definitions of many questionnaires in this area [23], this scale shall be based on a clear definition: Benefits are "positive aspects of informal caregiving" that have two characteristics:

1. The aspect is directly attributable to the informal caregiving activity ("Through the support/through the care of my relative/acquaintance …).
2. The aspect led to a personal enrichment, that means for the informal caregiver a "gain/added value" has resulted through the fact of giving care (e.g. "... I have become more patient").By considering these two aspects, we can speak of the concept of "benefits."

Furthermore, the goal of this study was to provide a quick and economical way to assess the personal “benefits” that can be experienced from caregiving. Such benefits can be influenced by interventions and can be validated for all CGs taking care of an older person at home. This validation of the Benefits of Being a Caregiver Scale (BBCS) includes investigations of internal consistency, factor analysis, discriminatory power and item difficulties, as well as convergent and discriminant validity.

## Methods

### Design

The data for the validation were obtained from the study “Benefits of Being a Caregiver.” Between October 2019 and March 2020, a total of 50 care assessors from the Medical Service of the Bavarian Health Insurance (MD) distributed 5,000 self-report questionnaires to statutorily insured informal CGs. These CGs applied for an initial grade or an increase in their CRs’ care level at the MD. Of the questionnaires we distributed, 1,082 (21.64%) were returned. By returning the completed questionnaire, the participants gave their consent to the anonymized use of the information they provided.

For the present study, approval was obtained from the ethics committee at the Friedrich-Alexander Universität Erlangen-Nürnberg (No.: 220_20 B).

### Sample

After excluding 121 cases because the CRs were younger than 65 years, the current sample for this validation included 961 cases. All CRs were living at home, and 52.8% were living with their CG. The mean age of the CGs was 62.10 years (*SD* = 12.6), 75.7% were female, 30.5% were spouses, 59.5% were caregiving children/-in-law or other CGs (e.g. aunts, uncles, nieces, nephews), and 47.8% were employed. The mean age of the CRs was 82.12 years (*SD* = 7.0), and 66.9% were female. A total of 37.9% of the CRs were receiving care because of dementia. Sample characteristics are given in Table 3.

### Instruments

#### Benefits of being a caregiver scale (BBCS) – scale development and description

The seven-step development of the BBCS included several methods: literature reviews, content analysis, surveys, and focus groups. The development was co-created by different experts and affected persons (researchers, care advisors, and caregivers). The development process is described in detail in Table 1. The final BBCS measures the benefits of being a CG at home with a 15-item self-assessment scale. The questionnaire was answered by the participants on a 5-point scale (4 = strongly agree, 3 = agree, 2 = neutral, 1 = disagree, 0 = strongly disagree). The score ranges from 0 to 60 points. Higher scores indicate greater CG benefits.

**Table 1:** Benefits of Being a Caregiver Scale – scale development

Steps	Goal	Methods/Participants	Results
1	Identify existing quantitative questionnaires evaluating PAC	Literature search (based on chapters of the Cochrane Handbook for Systematic Reviews of Interventions): Years of publication: 1990 and 2019 Languages: English and German - 2 master students guided by postdoc researcher	five questionnaires: Fabà, Villar, Giuliani, 2017; Yap Luo, et al., 2010; Tarlow Wisniewski, et al., 2004; Fulton Picot, Youngblut, Zeller, 1997; Strawbridge, 1991 eight subscales: Zank, Schack, Leipold, 2006; Farran, Miller, et al., 1999; Orbell, Hopkins, Gillies, 1993; Given, Given, et al., 1992; Schofield, Murphy, et al., 1997; Kinney, Stephens, 1989; Lawton, Kleban, et al., 1989; Motenko, 1989
2	Create a list of all items from the literature and combine the same or similar content	Analyze items for redundancies and similarities (according to Mayring, 2019) 2 master students guided by postdoc researcher and supervised by senior research advisor	- 143 items covering 12 different categories (e.g. personal development, meaning of life, …) - similar items from each category were named together (127 different aspects)
3	Compare the recorded items with qualitative statements (cited in the systematic review of Lloyd, 2016)	Analyze items for redundancies and similarities (according to Mayring, 2019) - 2 master students guided by postdoc researcher and supervised by senior research advisor	2 extra items covering new aspects (total 127 + 2 = 129 items)
4	Define the construct “benefits” - based on the results of the literature review and the current results on the topic of PAC	Focus group (according to Krueger, 2015) - 6 researchers and care advisors (a representative of the German Alzheimer´s society, health psychologist, nursing scientist, physician, health services researcher, psychologist)	all items on the new scale need to show (a) that family caregivers experience benefits for themselves and (b) the benefits they experience are due to their caregiving activities (“Caring for …”)
5	Identify the items (out of the 129 total items) that: - correspond to the definition of “benefits" - are important in the caregiving setting - can be influenced by interventions	Written survey (adapted from Flesch, 1948) and focus group to discuss the results of the survey - a majority decision had to be made (according to Krueger, 2015) - 6 researchers and care advisors (see above)	20 items
6	Analyze the 20 items for comprehensibility, redundancy, and similarities plus find the appropriate formulation format and scaling	Written survey (adapted from Flesch, 1948) - 6 researchers and care advisors (see above)	18 items every item includes: “Caring for my …” 5-point Likert scale ranging from 4 (strongly agree) to 0 (strongly disagree)
7	Analyze the 18 items for content, comprehensibility, and importance	Written survey (adapted from Flesch, 1948) - 5 informal caregivers	15 items (FINAL VERSION OF THE BBCS for the validation study)

### Further measurements

CGs’ subjective burden was measured with the 10-item short version of the Burden Scale for Family Caregivers (BSFC-s) [24]. Items are rated on a 4-point scale ranging from 0 (not true) to 3 (exactly true). Higher scores on the self-assessment scale indicate greater CG burden.

Positive aspects of caregiving were measured with the Positive Aspects of Caregiving (PAC) scale. The scale comprises 9 items that are rated on a five-point Likert scale ranging from 1 (strongly disagree) to 5 (strongly agree) [3]. Higher total sum values indicate that the rater experiences greater gains from the care situation.

CGs’ general coping behavior was assessed with the COPE 6 questionnaire, which was derived from the Brief COPE questionnaire [25]. The COPE 6 contains two items from each of the three scales problem-focused (“I've been concentrating my efforts on doing something about the situation I'm in “; “I’ve been trying to get advice or help from other people about what to do.”), emotion-focused (“I've been getting emotional support from others.“; “I've been getting comfort and understanding from someone.”), and avoidant coping (“I've been using alcohol or other drugs to make myself feel better.“;“I've been giving up trying to deal with it.”). The CGs were giving the following instruction: “The following statements relate to your thoughts and actions. How have you behaved in past unpleasant or difficult situations?” The items are evaluated on a 5-point scale ranging from 0 (strongly disagree) to 4 (strongly agree).

The relationship quality between the CG and CR was assessed currently and before the need for care with the following questions: "How would you rate the quality of the relationship between you and the person you support or care for?"/ "How would you rate the quality of the relationship between you and the person you support or care for before they needed your help or support?” The answer options were represented with a three-level pictorial response format, which characterized the quality of the relationship as "positive," "neutral," or "negative." Both items were subsequently dichotomized. Based on the assumption that socially desirable response behavior leads to negative relationships being classified as neutral [26], the categories "neutral" and "negative" were combined.

The three aspects of informal care time—ADLs (Activities of Daily Living), IADLs (Instrumental Activities of Daily Living), and supervision—were each evaluated with one item according to the Resource Utilisation in Dementia (RUD) questionnaire [27]. For each of the three aspects, the average daily number of hours spent giving care was also asked about. *ADLs* include activities such as dressing or personal hygiene. *IADLs* refer to activities such as preparing meals or taking medication. In addition, participants were asked whether and, if so, how many hours were needed for "supervision” (e.g. to avoid dangerous situations).

### Other measures

Sociodemographic and background characteristics were also assessed. These included, for example, CGs´ and CRs´ age and gender, CGs´ employment and educational attainment, the CG’s relationship to the CR (categorized into spouses and non-spouses), living situation (co-residing, yes or no), duration of care in months, reason for care (CR receives care because of dementia, yes or no), and CRs´ care level. All instruments were administered in German.

### Statistical analyses

#### Description

We calculated the mean, median, standard deviation, and distribution of the BBCS scores.

### Reliability and item analysis

We calculated Cronbach’s alpha as a measure of internal consistency. Cronbach’s alpha was calculated for the overall score. Bortz and Döring [28] recommended an alpha of 0.80 or higher as an indicator that a scale was well-designed. After the item analysis, the difficulty index and discriminatory power were calculated at the item level. While Bortz and Döring [28] recommended a corridor from 0.20 to 0.80 for the difficulty index, the discriminatory power was calculated as a deleted item-total correlation. According to Bortz and Döring [28], a discriminatory power of 0.30 to 0.50 can be classified as moderate and a power of > 0.50 as high.

### Factor analysis

To examine the underlying structure of the BBCS items, we performed an exploratory factor analysis. A scree plot depicts the distribution of the eigenvalues of the individual factors. We defined a factor loading ≥ 0.50 as the criterion for assigning a variable to a factor.

### Validity

The following hypotheses (H) were tested with regard to convergent (H1 – H3) and discriminant validity (H4):

- H1: Because the PACS and BBCS measure similar constructs, the two scales were expected to be strongly positively correlated [3].
- H2 : Some authors see a positive evaluation of benefits of being a CG as a meaningful coping resource [8], whereas others consider it to be a mediating variable [29]. However, many studies distinguish between adaptive (emotion-focused, problem-focused) and maladaptive (avoidant or dysfunctional) coping strategies [30]. In these studies correlations have demonstrated a small positive correlation between adaptive coping and PAC and no correlation between maladaptive coping and PAC (e.g. [31]). Therefore, CGs´ adaptive coping (emotion-focused, problem-focused) was expected to be rather positively correlated with the BBCS sum score (H2a) and maladaptive coping (dysfunctional) was expected to be not correlated with the BBCS sum score (H2b).
- H3: Different studies have shown a small positive association between a positive current relationship between the CG and CR and more positive aspects of care in daily life (e.g. [32, 33]). Therefore, a positive current relationship quality was expected to be positively correlated with the BBCS.
- H4: Initial results have indicated no association between subjective burden and the positive aspects of caregiving [12]. Thus, no correlation was expected between the BSFC-s and the BBCS.

To test H1, H2, and H4, the correlations between the BBCS score and the metric variables were computed as Pearson correlation coefficients (r). According to Döring and Bortz [34], correlations greater than 0.50 are considered strong, those between 0.30 and 0.50 are moderate, and those between 0.10 and 0.30 are weak. Correlations of less than 0.10 indicate that there is no association. In order to test H3, eta was calculated to identify the association between the BBCS score and the dichotomized relationship quality variable. According to Döring and Bortz, Eta^2^ greater than 0.14 are considered strong, Eta^2^ between 0.6 and 0.14 are moderate, and Eta^2^ between 0.01 and 0.05 are weak.

IBM SPSS version 28 for Windows was used for all statistical analyses. The cross-sectional baseline data were included in the analyses. A probability of error (alpha level) of 5% was set, below which statistical significance was indicated.

## Results

### Distribution of the BBCS scores

**Fig. 1:**
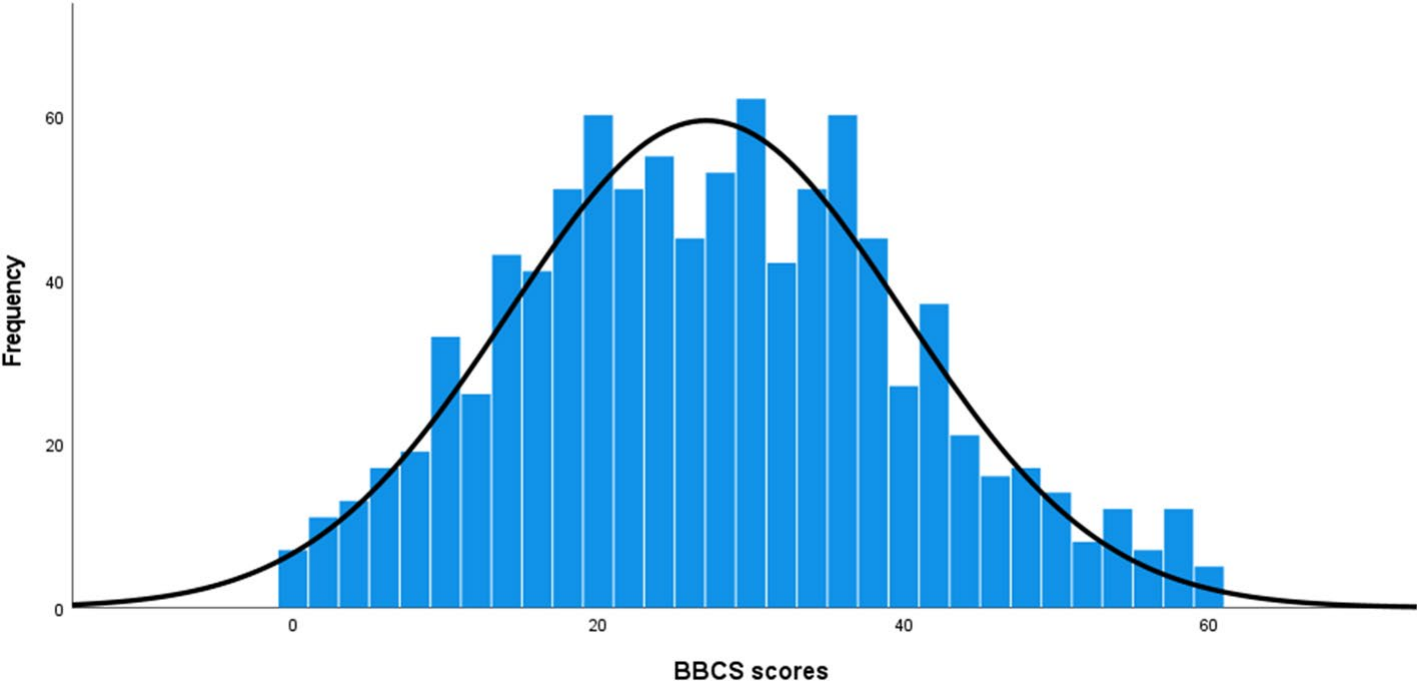
Distribution of the BBCS scores

The distribution of the BBCS scores in the present study ranged from 0 to 60 points (Fig. 1). Thus, the theoretically possible range of 0 to 60 points was completely exploited. The mean was 27.07 (*SD* = 12.91), and the median was 27.00.

### Item analysis and reliability

The difficulties of the BBCS items ranged from 0.28 to 0.67 (Table 2). The discriminatory power ranged from 0.45 to 0.74. No item showed a weak discriminatory power, and only the item “Through caring for my … I have met other caregivers who are important to me” had a moderate discriminatory power of 0.45. The other 14 items showed a high discriminatory power between 0.54 and 0.74. For the same 14 items, Cronbach’s alpha “if item deleted” was below 0.922, the alpha value for the whole scale (Table 2). Only the item with moderate discriminatory power had a Cronbach’s alpha “if item deleted” equal to 0.922. Because this item did not explain further variance in the benefits construct, and we aimed to develop a rather short scale, the item “Through caring for my … I have met other caregivers who are important to me” was deleted from the scale and from further analyses.

**Table 2:** Characteristics of the items of the BBCS

Item summary	Mean (SD)	Item diffi-culty	Discrimi-natory power	*p*	Cronbach’s α, “if item deleted”^a^	Factor loadings on factor 1
1) I have gotten to know myself better by caring for XX	1.80 (1.14)	.51	.64	< .001	.916	.70
2) Through caring for XX, I have become closer to her/him than before	1.90 (1.22)	.50	.59	< .001	.918	.64
3) Caring for XX has helped me organize my time better	2.12 (1.26)	.57	.58	< .001	.918	.64
4) I have the feeling that I have become more mature through caring for XX	1.65 (1.33)	.46	.69	< .001	.915	.75
5) Caring for XX has helped me to adopt a more positive attitude in life	1.49 (1.26)	.41	.71	< .001	.914	.76
6) Caring for XX has helped me to act more responsibly	1.97 (1.36)	.52	.74	< .001	.913	.79
7) Caring for XX has helped me to become more patient	1.90 (1.27)	.51	.62	< .001	.917	.68
8) Caring for XX has helped me to be a more understanding person	2.00 (1.22)	.53	.70	< .001	.915	.75
9) Caring for XX has helped me to be more aware of what values are important in life	2.59 (1.27)	.67	.62	< .001	.917	.69
10) Caring for XX has given my life more meaning	1.48 (1.26)	.40	.73	< .001	.914	.77
11) Caring for XX has strengthened the bonds between our family members and circle of friends	1.80 (1.28)	.47	.58	< .001	.918	.63
*Through caring for XX, I have met other caregivers who are important to me*^*b*^	*0.93 (1.13)*	*.28*	*.45*	< *.001*	*.922*	
12) Caring for XX has made me more self-confident in my relationships with other people	1.32 (1.22)	.38	.69	< .001	.915	.72
13) As a result of caring for XX, I feel more appreciated by other people	1.73 (1.24)	.49	.54	< .001	.919	.58
14) I have learned a lot more from caring for XX	2.40 (1.24)	.64	.64	< .001	.916	.69

*SD* standard deviation

*p*: p-value for discriminatory power

^a^Cronbach’s alpha (15 items) = .922

^b^item deleted for the final version of the BBCS

### Inter-correlations of the BBCS items

**Fig. 2:**
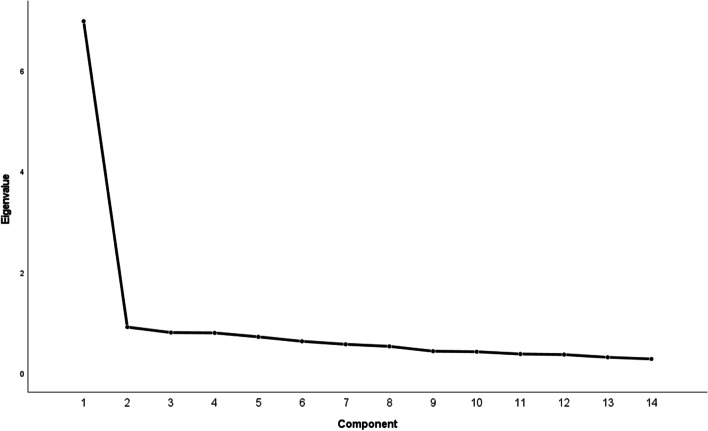
Factor analysis of the BBCS scores: scree plot

The exploratory factor analysis revealed one factor with an eigenvalue greater than 1.0 (6.9) (Fig. 2). This factor accounted for 49.8% of the total variance in the BBCS scores. All items had high loadings (factor loadings > 0.50) on this factor (Table 2).

### Convergent and discriminative validity

There was a significant positive association between the BBCS score and the PACS score (*r* = 0.75). The same trend was found between the BBCS score and better adaptive coping skills as well as a positive relationship between CG and CR: a higher BBCS score was observed when the CG had better emotion-focused coping (*r* = 0.18) and problem-focused coping (*r* = 0.23) skills or when the CG had a positive relationship with the CR (*η* = 0.20). The BBCS score was not associated with CGs’ subjective burden (*r* = -0.05) and dysfunctional coping (*r* = -0.07).

All correlations were in the expected direction and showed expected significant and not significant p-values (see Table 3). Consequently, all hypotheses related to the convergent and discriminant validity of the BBCS were supported.

**Table 3:** Construct validity of the BBCS’s hypotheses 1–4 and additional sample characteristics

Variable	*n (%)*	*M (SD)*	*r/ η*	*p*
Caregiver				
Age (years)		62.10 (12.63)	.10	.001
Sex (female)	727 (75.7%)		.03	.400
Employment (yes)	459 (47.8%)		-.14	< .001
Duration of care (months)		48.45 (78.72)	-.04	.253
Relationship (spouse, yes)	293 (30.5%)		.00	.940
Relationship quality before (positive)^*a*^	571 (59.5%)		.06	.220
*H1: Positive aspects of caregiving (PAC)*		*17.46 (9.20)*	*.75*	< *.001*
*H2Coping (COPE 6)*				
*H2a: emotion-focused coping scale*		*3.72 (2.22)*	*.18*	< *.001*
*H2a: problem-focused coping scale*		*4.04 (1.97)*	*.23*	< *.001*
*H2b: dysfunctional coping scale*		*6.15 (1.56)*	*-.07*	*.024*
*H3: Relationship quality actual (positive)*^*a*^	*554 (57.6%)*		*.20*	*.001*
*H4: Subjective care burden (BSFC)*		*16.71 (7.49)*	*-.05*	*.142*
Care-receiver				
Age (years)		82.12 (7.04)	-.09	.001
Sex (female)	643 (66.9%)		.03	.383
Dementia (yes)^*b*^	364 (37.9%)		-.06	.084
Level of care (2–5)^*c*^	632 (65.8%)		.04	.282
Care situation				
Co-residing (yes)	507 (52.8%)		.11	.001
ADL (h/d)^*d*^		2.69 (2.40)	.10	.003
IADL (h/d)^*d*^		3.45 (2.25)	.02	.582
Supervision (h/d)^*d*^		2.70 (3.25)	.09	.006
Informal help received (yes)^*e*^	576 (59.9%)		.03	.361

*M* mean*, SD* standard deviation, correlations with the BBCS sum score, *r* Pearson correlation *η* eta

*BSFC* subjective care burden (range 0–30), *COPE 6* coping (each scale range 0–8), *PAC* positive aspects of caregiving (range 9–45)

^*a*^relationship quality between the CG and CR (positive vs. neutral/negative)

^*b*^ CR receives care because of dementia

^*c*^The care level describes the extent to which care is needed on a 6-level ordinal scale: 0 (a little care needed)—5 (severe care needed). It is assessed by trained experts who are independent of the insurance system. Formal care is financed by long-term care insurance on the basis of the care level

^*d*^Consisting of hours per day (h/d) spent on activities of daily living (*ADL*), instrumental activities of daily living (*IADL*) and supervision of the CR by the CG

^*e*^CG receives informal support related to caregiving

## Discussion

The aim of developing a benefits scale was to "sharpen" the concept of benefits by including only items that represented a clear "gain" for family caregivers and could also be directly attributed to the home care situation as the cause of the gain. Special attention was paid to this aspect when selecting and formulating the items. In addition, not only did the scale need be suitable for the CGs of a person with dementia, as has been the case for most previous approaches [3, 5, 7], but it also needed to be valid for all other possible reasons a person might require caregiving in old age.

The 7-step development of the BBCS items began with a literature review in order to capture all aspects of benefits as comprehensively as possible as a starting point. The subsequent structured data reduction involved not only experts from different disciplines but also family caregivers so that the BBCS was developed in a "participatory" manner. This procedure maximized the validity of the content.

Fourteen of the 15 items contributed to the sum score (i.e. to the total extent of the benefits that were experienced) to a significant extent. Only for the item “Through caring for my … I have met other caregivers who are important to me” was it true that the internal consistency reliability of the total score (measured with Cronbach’s alpha) did not deteriorate if this item was deleted. However, the low difficulty of this item showed that CGshardly got to know other CGs through caregiving who were important to them. In terms of the psychosocial health of the CGs, however, it is important for exchanges between CGs to take place, as would be made possible, for example, by participation in a caregiver group. This aspect therefore remains an important aspect of counseling for CGs. However, it was not empirically significant for the BBSC sum score. We therefore decided to remove the item from the BBCS.

The hypotheses on convergent validity were supported, as shown by the high correlation between the BBSC sum score and the PACS score, which measures a similar construct. It is striking that the BBCS sum score could clearly be discriminated from the characteristics of the home care situation (e.g. the time spent each day assisting the CR with ADLs and IADLs) and from the socio-demographic parameters of the CGs as well as of the CRs (see correlations in Table 3). The BBSC had a quasi-zero correlation with the subjective burden construct. This lack of correlation means that these two constructs occur completely independently of each other in the home care situation. They are two different characteristics of the home care situation. Previous studies have already given indications of this finding [2].

### Strengths and weaknesses

This validation study was conducted on a large sample of family caregivers in Bavaria (Germany) with nearly 1,000 respondents who had applied for a care level for outpatient care or had applied for an increase in the care level. The empirically clearly evident one-factor structure of the BBCS shows that the sum score is justified and that this score can be interpreted as an expression of the scope of benefits. Moreover, the BBCS is not only applicable to home care situations involving dementia but to all situations that result in a need for caregiving among older people.

As a limitation, it must be noted that the validation sample was based only on relatives who were providing support at home in the sense of caregiving. Relatives who have to cover only minor needs for support were not included in the sample. Furthermore, the study sample consisted of self-selected German-speaking CGs who were able to complete the survey questionnaire independently, who might not be representative of the whole population of informal CGs. Additionally, this study was based on self-assessments and self-assessments are subject to various risks, e.g., responding is influenced by social desirability. This was not directly controlled in this study, but because most of the questionnaires were filled out anonymously, it can be assumed that it was less pronounced [35].

## Conclusions

There is a need for further research in the field of benefits. Future longitudinal or intervention studies should answer three research questions: What effects do interventions for family caregivers have on the caregivers’ experience of benefits? Which interventions for family caregivers can increase caregivers’ experience of benefits? If the benefits they experience increase or decrease, what impact do such changes have on the family caregiver, the care-receivers, and ultimately on the entire care situation?

The BBCS is a valid and time-efficient instrument for exploring these questions.

## Data Availability

The datasets used and analyzed during the current study are available from the corresponding author on reasonable request.

## Abbreviations

ADLs: Activities of daily living
BBCS: Benefits of Being a Caregiver Scale
BIZA: Berlin Inventory of Caregivers' Burden with Dementia Patients
BSFC-s: Burden Scale for Family Caregivers
CG: Caregiver
CR: Care receiver
GAC: Spanish Gains Associated with Caregiving Scale
IADLs: Instrumental activities of daily living
MD: Medical Service of the Bavarian Health Insurance
PAC: Positive Aspects of Caregiving
r: Pearson Correlation Coefficients
RUD: Resource Utilisation in Dementia
SD: Standard Deviation

## References

[1] García-Castro FJ, Alba A, Blanca MJ (2021). The role of character strengths in predicting gains in informal caregivers of dementia. Aging Ment Health.

[2] Pristavec T (2019). The burden and benefits of caregiving: A latent class analysis. Gerontologist.

[3] Tarlow BJ, Wisniewski SR, Belle SH, Rubert M, Ory MG, Gallagher-Thompson D (2004). Positive aspects of caregiving: Contributions of the REACH project to the development of new measures for Alzheimer’s caregiving. Res Aging.

[4] Lawton MP, Kleban MH, Moss M, Rovine M, Glicksman A (1989). Measuring caregiving appraisal. J Gerontol.

[5] Faba J, Villar F, Giuliani MF (2017). Development of a measure to evaluate gains among spanish dementia caregivers: The gains associated with caregiving (GAC) scale. Arch Gerontol Geriatr.

[6] Zank S, Schacke C, Leipold B (2006). Berliner Inventar zur Angehörigenbelastung-Demenz (BIZA-D). Z Klin Psychol Psychother.

[7] Yap P, Luo N, Ng WY, Chionh HL, Lim J, Goh J (2010). Gain in Alzheimer care INstrument–A new scale to measure caregiving gains in dementia. Am J Geriatr Psychiatry.

[8] Hudson P (2004). Positive aspects and challenges associated with caring for a dying relative at home. Int J Palliat Nurs.

[9] Pendergrass A, Mittelman M, Graessel E, Özbe D, Karg N. Predictors of the personal benefits and positive aspects of informal caregiving. Aging Ment Health. 2018;23(11);1533–38. 10.1080/13607863.2018.1501662 30428698

[10] Semiatin AM, O'Connor MK (2012). The relationship between self-efficacy and positive aspects of caregiving in Alzheimer's disease caregivers. Aging Mental Health.

[11] Schulz R, Belle SH, Czaja SJ, McGinnis KA, Stevens A, Zhang S (2004). Long-term care placement of dementia patients and caregiver health and well-being. JAMA.

[12] Lloyd J, Patterson T, Muers J (2016). The positive aspects of caregiving in dementia: A critical review of the qualitative literature. Dementia.

[13] Sanders S (2005). Is the glass half empty or full? Reflections on strain and gain in cargivers of individuals with Alzheimer's disease. Soc Work Health Care.

[14] Haley WE, Allen JY, Grant JS, Clay OJ, Perkins M, Roth DL (2009). Problems and benefits reported by stroke family caregivers: results from a prospective epidemiological study. Stroke.

[15] Picot SJF, Youngblut J, Zeller R (1997). Development and testing of a measure of perceived caregiver rewards in adults. J Nurs Meas.

[16] Strawbridge WJ: The effects of social factors on adult children caring for older parents. University of Washington; 1991.

[17] Farran CJ, Miller BH, Kaufman JE, Donner E, Fogg L (1999). Finding meaning through caregiving: Development of an instrument for family caregivers of persons with Alzheimer's disease. J Clin Psychol.

[18] Orbell S, Hopkins N, Gillies B (1993). Measuring the impact of informal caring. J Community Appl Soc Psychol.

[19] Given CW, Given B, Stommel M, Collins C, King S, Franklin S (1992). The caregiver reaction assessment (CRA) for caregivers to persons with chronic physical and mental impairments. Res Nurs Health.

[20] Schofield HL, Murphy B, Herrman H, Bloch S, Singh B (1997). Family caregiving: Measurement of emotional well-being and various aspects of the caregiving role. Psychol Med.

[21] Kinney JM, Stephens MAP (1989). Hassles and uplifts of giving care to a family member with dementia. Psychol Aging.

[22] Motenko AK (1989). The frustrations, gratifications, and well-being of dementia caregivers. Gerontologist.

[23] Kramer BJ (1997). Gain in the caregiving experience: Where are we? What next?. Gerontologist.

[24] Graessel E, Berth H, Lichte T, Grau H (2014). Subjective caregiver burden: validity of the 10-item short version of the Burden Scale for Family Caregivers BSFC-s. BMC Geriatr.

[25] Carver CS (1997). You want to measure coping but your protocol's too long: consider the Brief COPE. Int J Behav Med.

[26] Bogner K, Landrock U: Answer tendencies in standardized surveys. [Antworttendenzen in standardisierten Umfragen]. Gesis Survey Guidlines 2015:1–12. https://doi.org/12.10.15465/gesis-sg_016.

[27] Wimo A, Winblad B (2003). Resource utilization in dementia: "RUD Lite". Brain Aging.

[28] Bortz J, Döring N (2006). Forschungsmethoden und Evaluation: für Human- und Sozialwissenschaftler.

[29] Pakenham KI, Cox S (2018). Effects of benefit finding, social support and caregiving on youth adjustment in a parental illness context. J Child Fam Stud.

[30] Solberg MA, Gridley MK, Peters RM (2022). The factor structure of the brief cope: A systematic review. West J Nurs Res.

[31] Gardner MH, Mrug S, Schwebel DC, Phipps S, Whelan K, Madan-Swain A (2017). Demographic, medical, and psychosocial predictors of benefit finding among caregivers of childhood cancer survivors. Psycho-Oncol.

[32] Lum HD, Lo D, Hooker S, Bekelman DB (2014). Caregiving in heart failure: Relationship quality is associated with caregiver benefit finding and caregivber burden. Heart Lung.

[33] Tanji H, Anderson KE, Gruber-Baldini AL, Fishman PS, Reich SG, Weiner WJ, Shulman LM (2008). Mutuality of the marital relationship in Parkinson's Disease. Mov Disord.

[34] Döring N, Bortz J (2016). Forschungsmethoden und Evaluation in den Sozial- und Humanwissenschaften.

[35] Ong AD, Weiss DJ (2000). The impact of anonymity on responses to sensitive questions. J Appl Soc Psychol.

